# Keep Right: Inadvertent Placement of a Temporary Pacing Lead in the Left Ventricle

**DOI:** 10.7759/cureus.72095

**Published:** 2024-10-22

**Authors:** Dinesh Abhijeeth Shanker, Howard Briggs, Abraham Chacko

**Affiliations:** 1 Cardiology, Doncaster and Bassetlaw Teaching Hospitals NHS Foundation Trust, Doncaster, GBR

**Keywords:** 12-lead ecg, bundle branch block, cardiac arrythmia, pacing, st-elevation myocardial infarction (stemi)

## Abstract

Inadvertent lead malposition (ILM) of a temporary pacing wire is a rare complication that can occur during pacemaker insertion. We report a case of a 40-year-old man who presented late following an inferior ST segment elevation myocardial infarction. Temporary cardiac pacing was performed for symptomatic complete heart block. A 12-lead electrocardiogram (ECG) on the following day showed a paced QRS complex with right bundle branch block pattern. Malposition of the pacing wire was suspected to be in the left ventricle and confirmed by chest X-rays and an echocardiogram. This article discusses the occurrence of inadvertent left ventricle pacing and its management. Early recognition with the help of echocardiogram and ECG will aid in reducing the incidence of such complications.

## Introduction

Inadvertent lead malposition (ILM) of a temporary pacing wire is an infrequent complication [1,2]. It is difficult to know the actual incidence due to lack of reporting. In a retrospective study done by Ohlow et al., they noted an incidence of 0.34% [3]. Transvenous temporary pacing is usually indicated for symptomatic bradycardia that is expected to be reversible, while awaiting permanent pacing or when it must be delayed for recovery from other associated conditions such as an infection. The usual indications include acute inferior myocardial infarction (MI), bradycardia due to anti-arrhythmic drugs, post cardiac surgery, electrolyte imbalances and myocarditis due to acute infections such as Lyme disease.

In the absence of congenital heart disease, the right ventricle (RV) is the usual site for temporary pacing. ILM in the left ventricle (LV) can have major consequences. Recognising malposition of the temporary pacing wires requires a high index of suspicion. Electrocardiograms (ECG), chest X-rays, echocardiography and Computed Tomography (CT) scanning can assist in making a diagnosis [4].

We report a case of ILM of a temporary pacing wire in the LV. 

## Case presentation

**Figure 1:**
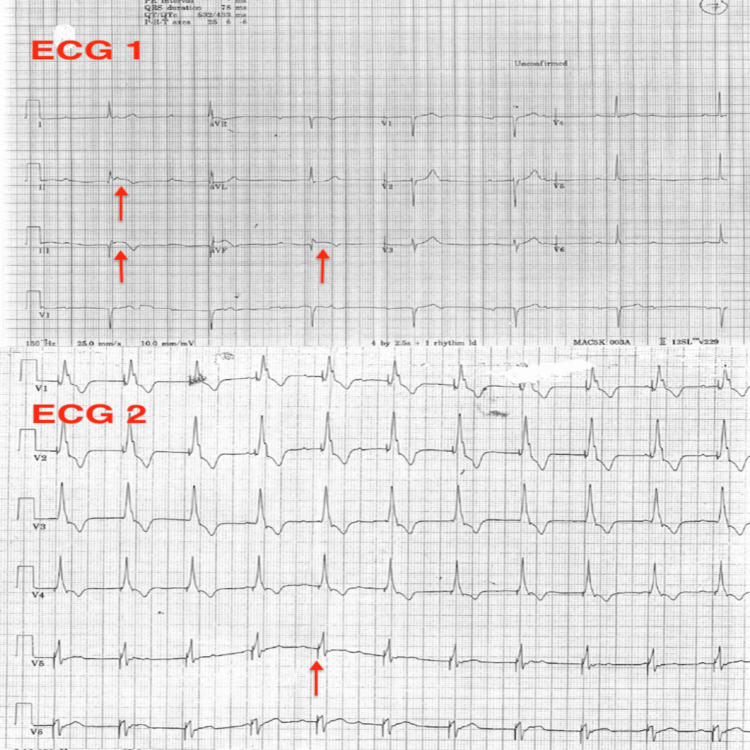
ECG 1: ECG at the time of presentation showing inferior MI (ST elevation marked by red arrows in lead II, III and aVF) and complete heart block. ECG 2: ECG demonstrating RBBB with pacing spikes (Pacing spikes marked by red arrow. Each pacing spike is followed by broad complexes in keeping with a right bundle brach pattern). MI: myocardial infarction, RBBB: right bundle branch block

A 40-year-old man, with no past medical or family history, presented 24 hours after developing chest pain. Initial evaluation with an ECG showed complete atrioventricular (AV) block, narrow QRS and ST segment elevation in leads II, III, aVF with Q waves and reciprocal ST depression in aVL in keeping with a recent acute inferior MI (ECG 1: Figure 1). Heart rate was 40/per minute, and the blood pressure was 110/70 mmHg.

Patient was managed as late presenting ST elevation MI. Since it was outside the reperfusion window, he was admitted to the coronary care unit for monitoring. Overnight, he developed further slowing of his heart rate, unresponsive to vagolytic agents and with blood pressure of 70/40 mmHg. A transvenous temporary pacing wire was placed via trans-venous approach. The procedure was uneventful, and the patient remained stable overnight. A post-procedure chest X-ray was not performed.

The next morning, the patient complained of intermittent dizziness that corresponded to intermittent loss of pacing on the cardiac monitor. A review of the 12 lead ECG showed right bundle branch block (RBBB) with pacing spikes (ECG 2: Figure 1).

**Figure 2:**
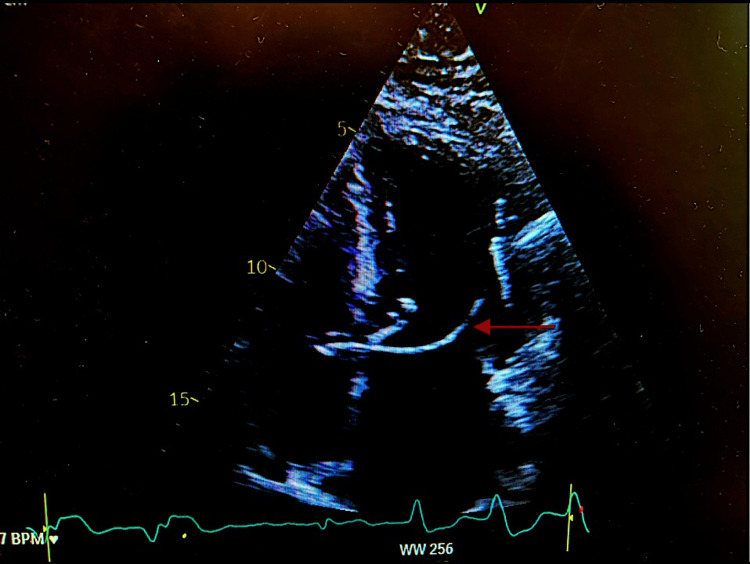
Four chamber view on transthoracic echocardiogram. Red arrow points to the temporary pacing lead traversing the interatrial septum and entering the left ventricle.

An urgent antero-posterior (AP) and lateral chest X-rays raised the suspicion of lead malposition in the LV. An echocardiogram showed that the pacing wire crossed the interatrial septum, entered the left atrium and then traversed across the mitral valve with the tip of the pacing lead in the LV (Figure 2).

Another temporary pacing wire was inserted via a trans-venous approach, into the right ventricle in an apical position. A chest X-ray was obtained before removal of the initial pacing lead. Post insertion ECG confirmed that the lead was in the RV as evidenced by left bundle branch block (LBBB) pattern. The patient made a full recovery with resumption of 1:1 AV conduction and the temporary pacemaker was removed. Repeat echocardiography did not demonstrate any shunt or pericardial effusion.

**Figure 3:**
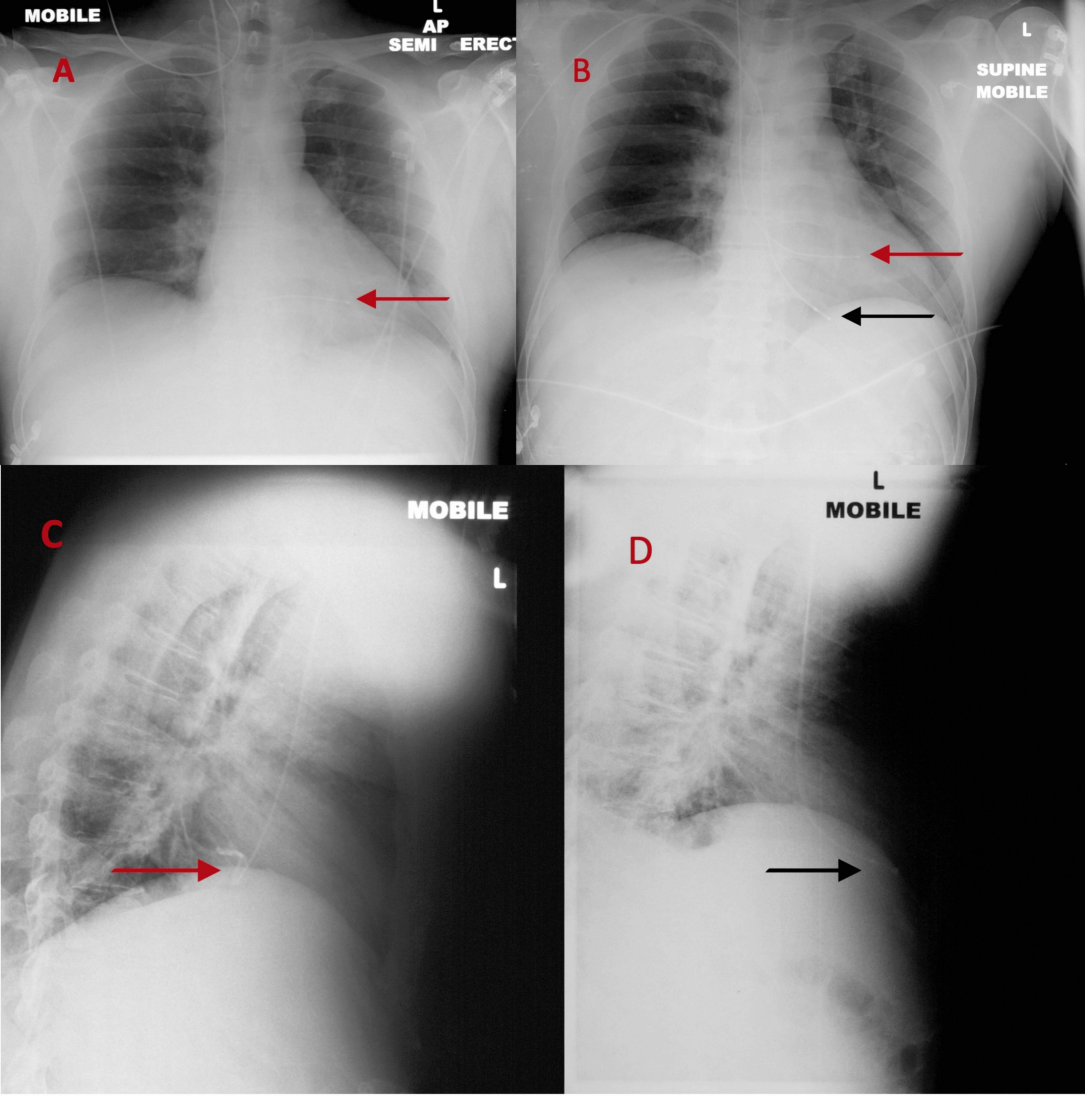
Chest X-ray AP Panel Views (A & B). Panel A: After initial pacing wire insertion, there is suggestion that the lead is in the LV. Panel B: After insertion of the second temporary pacing wire, panel B shows both leads in distinctly different positions. Chest X-ray Lateral Panel Views (C & D). C: Shows position of the first temporary wire directed posteriorly toward the spine (in LV). D: Shows lead positioning of the second wire anteriorly in the RV away from the spine. Note that the initial temporary wire had been removed. Black arrow- RV lead. Red arrow- LV lead. AP: antero-posterior, LV: left ventricle, RV: right ventricle

**Figure 4:**
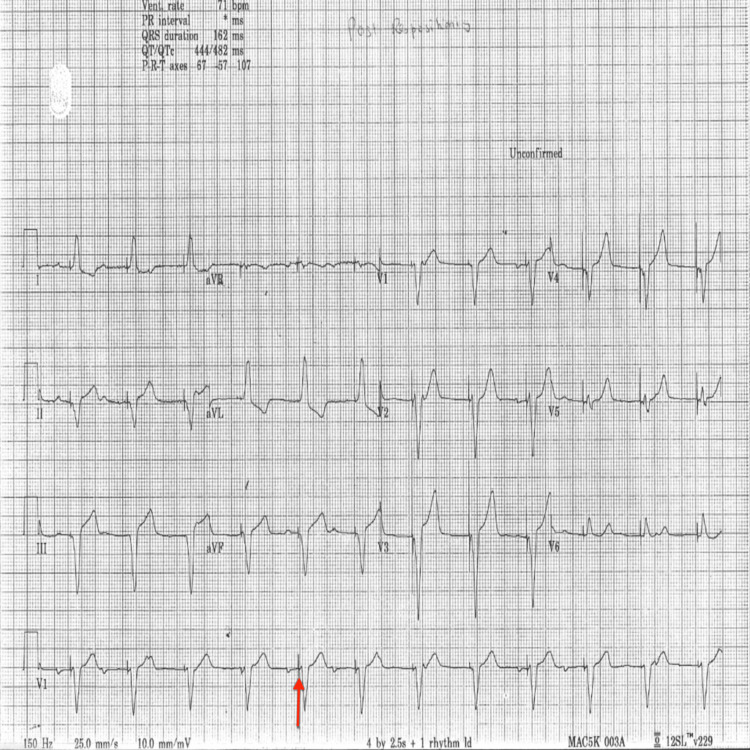
ECG following insertion of the RV lead and after removal of the LV lead. Arrow points to the pacing spike followed by broad QRS complex which is in keeping with LBBB. LV: left ventricle, RV: right ventricle, LBBB: left bundle branch block

Figure 3 shows the X-ray images before and after the insertion of the second wire to demonstrate the positions of right and left ventricular leads. Figure 4 shows ECG following insertion of the RV lead and after removal of the LV lead.

## Discussion

We report a case of ILM of a temporary pacing wire in the left ventricle due to the lead traversing the interatrial septum and highlight the importance of a 12 lead ECG and an AP and a lateral views of chest X-ray after the procedure to confirm correct lead positioning. RV pacing typically results in an LBBB pattern in lead V1. An RBBB pattern in lead V1 after endocardial pacing should raise the suspicion of lead malposition in the LV or an LV branch of the coronary sinus. Rarely, a very apical position of the lead in the RV can be associated with an RBBB pattern due to a wrap-around RV chamber or due to apical septal pacing.

The major complication relating to lead placement in the LV is the risk of thrombosis and systemic embolism [3,5,6]. Spighi et al. in their systematic review, noted transient ischemic attacks or strokes in a third of cases [3]. Other potential adverse effects include mitral valve leaflet perforation and/or mitral regurgitation [6].

ILM into the LV can occur due to cannulation of an artery instead of the vein, traversing a patent foramen ovale (as likely in this case) or septal defects. An important aspect of right heart catheterization for temporary pacing via internal jugular vein or subclavian vein, is to place a J-tipped guide wire through the superior vena cava and right atrium and visualize its passage through the inferior vena cava (right side of the spine) as opposed to passage to the descending aorta which courses left of the spine in case of inadvertent arterial puncture. Complications relating to inadvertent cannulation of the arterial system can include damage to the coronary arteries and aortic valve in addition to the thromboembolic complications noted above.

Initial clues for malposition may be picked up on an ECG by the occurrence of a paced RBBB pattern in V1 [7]. It is important to be aware that not all RV pacing results in LBBB. For instance, early penetration of the electrical signals into LV in an apical RV lead may result in RBBB. RBBB with left superior axis deviation or qR or RS morphology in V1 is quite specific and sensitive for RV pacing [8]. RBBB morphology may be seen in coronary sinus pacing [8]. Klein’s manoeuvre may help to differentiate between the RBBB from an LV pacing lead and a pseudo-RBBB due to an appropriately positioned RV pacing lead [9]. By shifting the V1 and V2 leads to the 5th intercostal space, if the RBBB changes to an LBBB, then it may be concluded that the pacing lead is in the RV. However, if the RBBB persists despite this manoeuvre, ILM in the LV should be suspected [5,8,10].

Chest X-ray also plays an important role in lead position assessment. In an AP view, the LV lead is more superior and displaced towards the left while in the lateral view, LV lead is facing posteriorly towards the spine [3,5]. On the contrary, the RV lead traverses through the right atrium and the tricuspid valve into the RV with a slight bowing giving it a ‘ballerina foot’ appearance in the AP view and in the lateral view, RV lead faces anteriorly [3].

Echocardiography can delineate the path of the pacing wire in the heart chambers (Figure 3). It will also help to assess the presence of mobile masses representing thrombus. If transthoracic echocardiogram (TTE) is inconclusive, transoesophageal echocardiogram (TOE) may be considered.

ILM in the LV can be easily removed and repositioned if recognized early. If there is a suspicion of thrombus on the lead, management becomes more complex and may require the use of carotid protection devices prior to removal. This is more common with permanent pacing leads implanted in the LV. Thrombus may develop as early as two weeks [11]. Options in such situations include long-term anticoagulation or removal at surgery. While there are reports of extraction of permanent pacemakers after nine months of insertion, these procedures come with risks [3]. ALSNYC trial noted that despite an international normalised ratio (INR) of 3, 11% of the patients with a permanent pacing lead placed in the LV developed thromboembolic events while on warfarin [5]. In a meta-analysis done by Dalia et al., direct oral anticoagulants (DOAC) was found to be non-inferior to warfarin without any significnat increase in bleeding complications [12]. If the patient is undergoing cardiac surgery for another indication, extraction may be attempted during the surgery [3,5,13]. In these cases, the surgical risks must be weighed against that of chronic anticoagulation with the lead left in situ.

## Conclusions

ILM resulting in LV pacing is a rare complication that occurs after temporary or permanent pacing that can result in serious complications such as thromboembolic events. ECG, chest X-rays and echocardiogram will aid in diagnosis. Prompt recognition is the key to early removal and repositioning to avoid complications.
